# Expression and Functional Analysis of the Argonaute Protein of *Thermus thermophilus* (TtAgo) in *E. coli* BL21(DE3)

**DOI:** 10.3390/biom11040524

**Published:** 2021-03-31

**Authors:** Jiani Xing, Lixia Ma, Xinzhen Cheng, Jinrong Ma, Ruyu Wang, Kun Xu, Joe S. Mymryk, Zhiying Zhang

**Affiliations:** 1 Key Laboratory of Animal Genetics, Breeding and Reproduction of Shaanxi Province, College of Animal Science and Technology, Northwest A&F University, Yangling 712100, China; jianixing@nwafu.edu.cn (J.X.); datong19861125@163.com (L.M.); Chengxz0816@163.com (X.C.); dongkemjr@163.com (J.M.); wry1719@163.com (R.W.); xukunas@nwafu.edu.cn (K.X.); 2 Central Laboratory, Changzhi Medical College, Changzhi 046000, China; 3 Department of Microbiology & Immunology, Oncology and Otolaryngology, The University of Western Ontario, London, ON N6A 3K7, Canada; jmymryk@uwo.ca

**Keywords:** TtAgo, in vivo, endonucleases, mutagenesis, *fabI*, triclosan

## Abstract

The prokaryotic Argonaute proteins (pAgos) have been reported to cleave or interfere with DNA targets in a guide-dependent or independent manner. It is often difficult to characterize pAgos in vivo due to the extreme environments favored by their hosts. In the present study, we expressed functional *Thermus thermophilus* pAgo (TtAgo) in *E. coli* BL21 (DE3) cells at 37 °C. Initial attempts to express TtAgo in BL21(DE3) cells at 37 °C failed. This was not because of TtAgo mediated general toxicity to the host cells, but instead because of TtAgo-induced loss of its expression plasmid. We employed this discovery to establish a screening system for isolating loss-of-function mutants of TtAgo. The *E. coli* *fabI* gene was used to help select for full-length TtAgo loss of function mutants, as overexpression of *fabI* renders the cell to be resistant to the triclosan. We isolated and characterized eight mutations in TtAgo that abrogated function. The ability of TtAgo to induce loss of its expression vector in vivo at 37 °C is an unreported function that is mechanistically different from its reported in vitro activity. These results shed light on the mechanisms by which TtAgo functions as a defense against foreign DNA invasion.

## 1. Introduction

Argonaute proteins are present in all three domains of life and are the subject of much recent attention for their ability to bind a nucleic acid guide sequence and subsequently cleave single-stranded DNA or RNA targets in vitro [1,2,3,4,5], interfering with DNA integrity [6] and gene expression [7]. They can also enhance homologous sequence-directed recombination by interacting with RecA [6], and play roles in DNA replication in vivo [7]. Unlike eAgos, which have been intensively studied for their role in the RNA interference (RNAi) pathway [8,9], prokaryotic argonaute proteins (pAgos) are much more diverse in classification and behaviors in different hosts. Since archaea and bacteria lack RNAi pathways [10], and some pAgo genes are found close to CRISPR loci [11], their ability to degrade nucleic acids [1,2,3,4,5,12], interfering with DNA [13,14] and RNA targets [12,15,16,17], suggested that they may serve as a defense mechanism for archaea and bacteria against parasitic nucleic acids [18,19]. This also makes them potentially attractive new tools for gene-editing tool research [20].

By mining the expanding repertoire of microbial genome sequences, many pAgo genes have been reported, such as *Thermus thermophilus* (TtAgo) [4,12,21], archaeon *Pyrococcus furiosus* (PfAgo) [1,22], archaeal organism *Methanocaldococcus jannaschii* (MjAgo) [5,23,24], CRISPR-associated *Marinitoga piezophila* (MpAgo) [11,25,26], *Clostridium butyricum* (CbAgo) [2,13,14,27], *Rhodobacter sphaeroides* (RsAgo) [28,29], and *Natronobacterium gregoryi* (NgAgo) [15,16]. These pAgos have been partially characterized and demonstrated to function as nucleases with different properties in terms of guide-binding and target-processing. Most of the published pAgo research has been focused on protein structure and enzyme activity using in vitro cleavage assays [2,5,14,27,30], with limited data from in vivo studies [7,12,13,15,16,28]. As such, detailed mechanisms of their function remain elusive.

Enzymatically dead pAgo is often used as an experimental control in pAgo studies, both for characterizing structural changes and enzyme activity studies. Sequence alignments between pAgos and eAgos identified a conserved DEDX (X = N, D, or H) tetrad catalytic center in many long pAgos, including TtAgo, MjAgo, MpAgo, PfAgo, CbAgo, and NgAgo, necessary for cleaving nucleic acid targets [2,3,4,5,12,23,24,26,28,29,31,32,33]. Based on this conserved nuclease activity domain, it is assumed that an enzymatically dead mutant pAgo protein can be easily generated by mutating the DEDX motif. Mutations in some pAgos that change the glutamate finger conformation, and their contact with the active center, influence the binding of metal ions and block enzyme activity [30,34,35]. Additionally, mutations in the DEDX active center of TtAgo [4,31], MpAgo [25,26], MjAgo [5], PfAgo [1], and CbAgo [2,27] abrogate enzymatic activity measured by in vitro cleavage assays. Furthermore, no small guide DNAs (smDNAs) were generated by a TtAgo double mutant (TtAgoDM; D478A, D546A) [4].

In contrast to in vitro studies, results obtained from in vivo experiments suggest that mutants in the DEDX active center retain at least some function. Specifically, although the CbAgo double mutant (CbAgoDM; D541A, D611A) exhibits a weaker activity for binding smDNAs and a smaller but still significant effect on defending against M13 phage infection, it retains some function [13]. In addition, the NgAgo single mutant (D663A or D738A) and double mutant (NgAgoDM; D663A, D738A) were reported to be capable of causing abnormal eye phenotype in zebrafish [15], indicative of retention of function. Thus, several pAgos mutants have been reported to behave differently when tested in vivo and in vitro. These observations suggest that pAgos may function as a host defensive system differently in vivo than described in vitro. However, the use of homologous systems to study the pAgo can be quite difficult, including extreme growth conditions or the lack of well-established plasmid expression and selection systems. This prompted us to establish an in vivo model using *E. coli* strains to study the mechanisms of pAgos function in vivo.

Almost all of the pAgos have been reported to be expressed poorly in *E. coli* BL21 and its derivatives [1,2,4,13,25,31]. Some studies report obtaining recombinant pAgo protein at a lower temperature (16 °C or 18 °C), but not at 37 °C [2,25]. A general toxicity of pAgos to the host cell has been blamed for these failures, including affecting bacterial cell growth, but the exact reason for toxicity remains to be investigated. Because of the difficulty of expressing pAgos, no *E. coli* cell model has been established to mechanistically study pAgos function in vivo.

Triclosan is a Food and Drug Administration (FDA)-approved non-antibiotic biocide agent found in many products, including toothpaste, soaps, shampoos, and other kinds of cleaning products [36,37]. Triclosan binds to bacterial enoyl-acyl carrier protein reductase enzymes (ENRs). In *E. coli*, the target ENR is encoded by the *fabI* gene, and triclosan inhibits FabI by binding at the CAP-enoyl substrate site, forming an inactive triclosan-NAD^+^/FabI complex. Overexpression of *fabI* in *E. coli* confers resistance to growth inhibition growth by triclosan [38]. Thus, the *fabI* gene can potentially be employed as a new marker gene with utility in high throughput screens.

In the present study, we first investigated why TtAgo failed to be expressed in *E. coli* BL21 cells grown at 37 °C. We employed different strategies to confirm that TtAgo was not a general toxin to host cells. Surprisingly, we discovered that plasmid-expressed TtAgo induces loss of its expression vector and we also found that fusing a His-tag at 3′end of TtAgo coding sequence does not abolish this activity of TtAgo protein. We leveraged this discovery to develop a high-throughput screening system for isolating loss-of-function TtAgo mutants in vivo that relies on fusing the *fabI* gene with *TtAgo*. By screening a library of random mutations in *TtAgo* generated by error-prone PCR (EP-PCR), we successfully isolated eight loss-of-function mutants with a single-point mutation of TtAgo. Our data lays the foundation for investigating the in vivo functions of pAgo proteins in their host cells.

## 2. Materials and Methods

### 2.1. Strains and Cultivation

The *E. coli* BL21(DE3) strain was used for electroporation and protein expression throughout the study. The *E. coli* DH5α strain was used to maintain the plasmids and for vector construction. Bacterial cells were cultured in standard Luria-Bertani broth (1% (*w*/*v*) NaCl, 1% (*w*/*v*) Tryptone, 0.5% (*w*/*v*) Yeast Extract, pH 7.0) with the addition of the appropriate antibiotics at 37 °C. Rich medium SOC (2% (*w*/*v*) Tryptone, 0.5% (*w*/*v*) Yeast Extract, 10 mM NaCl, 2.5 mM KCl, 10 mM MgCl_2_, 20 mM Glucose, pH 7.0) was used for bacterial cell recovery after electroporation.

The final concentration of kanamycin was 50 μg/mL, inducer L-arabinose (Solarbio^®^, Beijing, China, L8060) was 10 mM, inducer isopropyl β-D-thiogalactopyranoside (IPTG) was 0.5 mM, and triclosan (Macklin, Shanghai, China, #C10101458) was tested and determined to be 1 μM.

### 2.2. Construction of TtAgo Expression Vector pET28a-TtAgo and pET28a-pBAD-TtAgo

The TtAgo coding sequence (GenBank ID: AP008227) was synthesized and was cloned in *Eco*RV site in the pUC57-simple vector by ShineGene Molecular Biotech, Inc. (Shanghai, China). Primers *Nde*I-Tt-F and *Hin*dIII-TAA-TtV685-R were used to amplify the TtAgo coding sequence and cloned into the pET28a vector (kept by our laboratory) between *Nde*I and *Hin*dIII, thus the construct contains 6 × His tag and thrombin-site in the N-terminus, named pET28a-TtAgo. Primers *Sal*I-Tt-F and *Hin*dIII-6 × His-Tt-R were used to add another 6 × His tag in the C-terminus of TtAgo coding sequence, generating an *E. coli* expression vector with a 6 × His tag at both ends of *TtAgo* gene, named pET28a-TtAgo-His tag.

We noticed that there was a degree of leaky expression for T7 promoter-driven expression in *E. coli* BL21(DE3). In order to increase the sensitivity and efficiency of high-throughput screening for loss-of-function mutants of TtAgo, we chose the araC/pBAD system because it was more tightly regulated [39]. This promoter switch was based on the functional comparison between TtAgo protein driven by T7 and araC/pBAD promoters. The TtAgo protein expressed by araC/pBAD has the same activity and a similar yield as that expressed by T7 promoter. This promoter switch further confirmed the pAgo interference with the plasmids.

An *araC* gene with pBAD promoter sequence was amplified from plasmid pKD46 (kept by our laboratory) with primers *Bgl*II-araC-R and *Xba*I-pBAD-R and then cloned into pET28a-TtAgo-His tag between *Bgl*II and *Xba*I sites to replace the original sequence. The vector was named as pET28a-pBAD-TtAgo and the primers used were listed in Supplementary Table S1.

pET28a-NgAgo plasmid was kindly given by Dr. Tingting Zhang from Shanxi University, China, and pET28a-RsAgo plasmid was generously given by Dr. Miyoshi from Niigata University, Japan [29].

### 2.3. FabI-Fusion Construction

The pre-existing *Xba*I site in the pET28a-pBAD-TtAgo vector was removed with the primers *Eco*RI-*Xba*I del-F and *Nde*I-*Xba*I-del-R in preparation for *fabI* gene cloning. *FabI* gene was amplified from *E. coli* BL21 (DE3) genomic DNA with the primers *Spe*I-gsg link-fab1-F and *Xba*I-fab1-R. A single amino acid mutation G93V in its coding sequence was introduced with primers FabIG93V-overlap-F and FabIG93V-overlap-R and was cloned downstream of the *TtAgo* gene in pET28a-pBAD-TtAgo between *Spe*I and *Xba*I for a better and more efficient interaction with triclosan [40,41]. The stop codon of the *TtAgo* gene was replaced with a linker sequence encoding the amino acids GSG and included an extra G nucleotide to make the *TtAgo* gene and *fabI* gene in the same open reading frame. This chimeric gene is under control of the promoter pBAD, which provides tighter control of expression compared to IPTG induction from the T7 promoter [39]. The primers used are listed in Supplementary Table S1. The newly constructed plasmid was named pET28a-pBAD-TtAgo-fabI. A control plasmid expressing a defective TtAgo was generated by cleaving wild-type TtAgo sequence with *Sac*I and then self-ligation. The truncated TtAgo mutant was confirmed to be enzymatically inactive since partial of N-term and MID domain was cut off and the plasmid was not lost after induction and was named pET28a-pBAD-ΔTtAgo-fabI.

### 2.4. Construction of TtAgo Random Mutation Library by Error-Prone PCR (EP-PCR)

To enhance the mutagenesis efficiency, we first optimized the EP-PCR conditions including concentration of Mg^2+^, and dNTPs in the PCR reaction system. The full-length TtAgo sequence was amplified with the primers *Nde*I-Tt-F and *Spe*I-Tt-R (Supplementary Table S1). The PCR products generated from different conditions were directly sequenced and the mutagenesis efficiency was scored based on the number of double-peaks detected by sequencing. The optimal EP-PCR conditions that yielded the highest mutagenesis efficiency included 5 mM of MgSO_4_, 0.5 mM of MnCl_2_, 0.2 mM of dGTP, 1 mM of dATP, dCTP, and dTTP (Supplementary Table S2). The MnCl_2_ was added in the last step when preparing the reaction. The EP-PCR reaction was conducted in 50 μL and started at 94 °C for 10 min, and then ran 35 cycles of 94 °C for 1 min, 58 °C for 1 min, and 72 °C for 5 min, and finally extended 10 min at 72 °C.

### 2.5. Construction and Selection for Loss of Enzyme Function Mutants

The TtAgo sequence in pET28a-pBAD-TtAgo-fabI was replaced by EP-PCR amplification products through double enzyme digestion (*Nde*I/*Spe*I) and T4 ligation. The ligation products were directly purified using a PCR extraction kit (Omega Bio-Tek, Guangzhou, China) following the manufacturer’s instruction. The purified EP-PCR ligation products were directly electroporated into *E. coli* BL21 (DE3) competent cells. Transformed cells were recovered in 1 mL of SOC liquid medium for 1 h. Ten microliters were plated on LB plates containing kanamycin to determine the electroporation efficiency, and the rest of the cells were plated on selection plates containing kanamycin, L-arabinose, and triclosan to identify loss-of-function mutants. The selection plates were placed to avoid light and grown for more than 20 h. Single colonies were picked and used to inoculate fresh LB liquid medium containing kanamycin and cultivated in a shaker incubator at 37 °C overnight for plasmid recovery (Plasmid mini kit, Omega Bio-Tek, Guangzhou, China). The recovered plasmids were used to retransform the BL21(DE3) for loss-of-function phenotype confirmation. The confirmed plasmids were sent for Sanger sequencing (TSINGKE, Beijing, China).

### 2.6. Construction of Single Point Mutations Isolated from Screening

Eight individual point mutations derived from the identified 42 TtAgo mutants were selected for further analysis and the locations of the mutations are summarized in Table 1. To generate these point mutations de novo, site-specific mutagenesis primers were synthesized with the mutated nucleotide in the middle of the forward and reverse primers (TSINGKE Company, Xi’an, China) and are listed in Supplementary Table S1, and the re-constructed plasmids are listed in Table 2.

### 2.7. Construction of the TtAgo DEDX Mutants by Site-Directed Mutagenesis

Site-directed mutagenesis primers were designed to alter the TtAgo DEDX site. Primer sequence are listed in Supplementary Table S1. This in vitro dead double mutant of TtAgo was designated as TtAgoDM (D478A, D546A) and re-constructed in pET28a-pBAD-TtAgo between *Sal*I and *Hin*dIII. The new plasmid was named as pET28a-pBAD-TtAgoDM. Mutants within with two symmetrical arginine mutations (R486H and R545H) were similarly constructed using primers R486-overlap-F/R and R545-overlap-F/R and similarly cloned into pET28a-pBAD-TtAgo between *Sal*I and *Hin*dIII. These two were named as pET28a-pBAD-TtAgo (R486H) and pET28a-pBAD-TtAgo (R545H). All mutations were confirmed by Sanger sequencing (TSINGKE, Xi’an, China).

### 2.8. Spot Plating Assay

After transformation of TtAgo expression vector and derived mutants, 3 single colonies were picked from each plate and inoculated into LB liquid medium containing kanamycin and allowed to grow in a shaking incubator at 37 °C until the OD_600_ approached 0.5 (~2.5 × 10^8^ CFU). Cells were diluted to 10^−2^, and then serial 100-fold dilutions were made until 10^−6^. Five microliters of each dilution were spotted on the LB plate supplemented with or without antibiotics and promoter inducers as designated for specific experiments. The plates were then incubated at 37 °C and growth was examined for 24 h.

The main purpose of the spot test is to provide a visible example of the effect of pAgo expression on cell growth under the defined conditions. We have tried a different number of input cells and series of dilutions to achieve a clear growth spot for the control group. The cell density numbers labelled on the figures were based on our optimized calculations, and it is hard to avoid the possibility of some cell number variation caused by the operation process.

### 2.9. Protein Expression

pET28a-pBAD-TtAgo and the TtAgo mutant expression plasmids were transformed into *E. coli* BL21 (DE3) competent cells, and colonies cultivated in 2 mL LB medium containing kanamycin in a shaking incubator at 37 °C overnight. Five hundred microliters of saturated cells were added into 9.5 mL LB medium containing antibiotic. These were cultivated with shaking at 37 °C for about 2.5 h until an OD_600_ of 0.45–0.55 was reached. Protein expression of TtAgo and their mutants were induced by adding L-arabinose. Induction was continued in a shaking incubator at 37 °C for 4 h and the cells were collected by centrifugation. Cell pellets were washed with cold ddH_2_O once and resuspended in 400 μL lysis buffer (50 mM Tris-HCl, 150 mM NaCl, 5% glycerol, pH 8.0) supplemented with 1 mM PMSF, then disrupted by sonication with a handheld sonication machine (Hielscher, UP50H, Germany; purchased from Labsun, China) with a 5 mm tip at a medium setting (60% amplitude with a pause with 0.5 s) on ice to reduce heat denaturation. One-hundred-microliter samples were mixed with 25 μL 5 × SDS-PAGE Loading Buffer (250 mM Tris-HCl, pH 6.8, 10% (*w*/*v*) SDS, 0.5% (*w*/*v*) BPB, 50% (*v*/*v*) glycerol, 5% (*v*/*v*) 2-ME), and boiled in hot water for 10 min, and were centrifuged for 5 min at 10,000× *g* at room temperature before gel loading.

### 2.10. Western Blotting

Protein samples mixed with 5 × SDS-PAGE Loading Buffer were resolved by electrophoresis in a 10% SDS-PAGE gel (Solarbio^®^,Beijing, China). Proteins were transferred onto a PVDF membrane in transfer buffer (39 mM glycine, 48 mM Tris, 0.037% (*w*/*v*) SDS, 20% (*v*/*v*) methanol) using a wet procedure at 300 mA for 1 h. The PVDF membrane was blocked with blocking buffer (5% (*w*/*v*) non-fat milk dissolved in TBST buffer (20 mM Tris-HCl, 150 mM NaCl, 0.05% (*v*/*v*) Tween 20)) at room temperature for at least 1 h and then washed with TBST buffer 3 times and incubated with His-Tag Mouse Monoclonal antibody (1:10,000, Proteintech, Cat# 66005-1-Ig) for 1.5 h at room temperature. The membrane was washed for 10 min with TBST buffer on an orbital shaker. This was repeated for two additional washes and then the blot was incubated with Goat anti-mouse secondary antibody (1:5000, CWBIO, Cat# CW0102S) at room temperature for 1 h, and washed with TBST as described above. Protein expression was detected with ECL Western HRP substrate (Advansta) on a Fluorescent gel imaging system (MicroChemi, DNR).

### 2.11. Protein Structure Simulation

The structure of the TtAgo protein in apo form was simulated using SWISS-MODEL (https://swissmodel.expasy.org; accesed on 11 March 2021) through the Homologous Modeling method based on the template in the SWISS-MODEL [42,43,44,45,46](ID: 4nca.2, Structure of Thermus thermophilus Argonaute bound to guide DNA 19-mer and target DNA in the presence of Mn2+) [35]. The structure of TtAgo mutants was simulated with VMD software (Visual Molecular Dynamics, Theoretical and Computational Biophysics Group, http://www.ks.uiuc.edu/Research/vmd/; accesed on 11 March 2021), using the wild-type TtAgo structure as the template to introduce mutations. In addition, the mutation sites were labeled with VMD software.

## 3. Results

### 3.1. Expression of pAgo Protein in E. coli BL21 (DE3)

As previously reported, several pAgo genes failed to be expressed in E. coli BL21(DE3) strain at 37 °C, presumably due to their general toxicity to the host cells [14,25,31]. To investigate how pAgo proteins exert their toxicity on heterologous host E. coli BL21(DE3) cells, we cloned the TtAgo (Thermus thermophilus, GenBank ID: AP008227) gene into the E. coli expression vector pET28a (designated as pET28a-TtAgo), which allows IPTG regulated expression, and transformed BL21(DE3) cells. We examined the effects of its expression on both host cell growth and vector yield.

**Figure 1:**
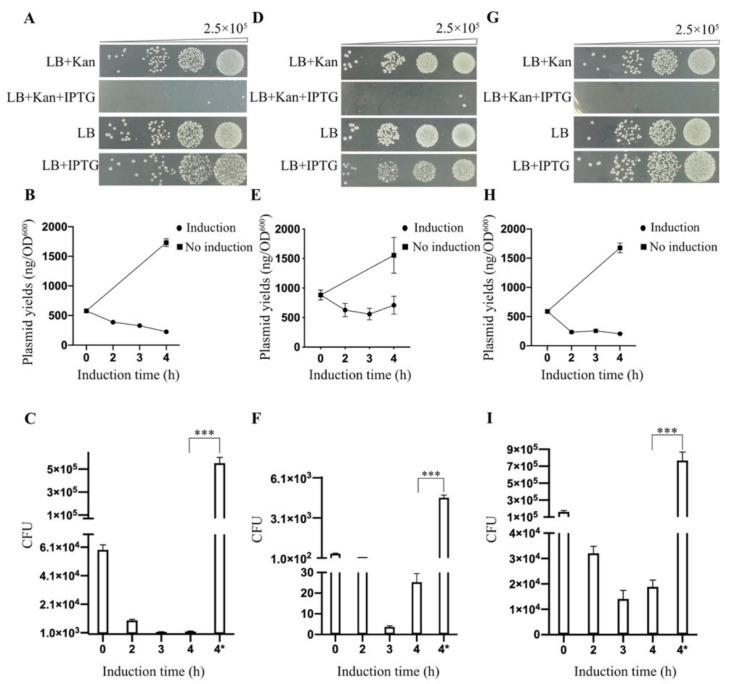
Expression of prokaryotic Argonaute proteins (pAgos) in *E. coli* BL21(DE3). (**A**) Spot plating assay for the effect of TtAgo expression on the host cell genome and its expression vector degradation. Cells were grown with or without isopropyl β-D-thiogalactopyranoside (IPTG) as noted. The upper panels were grown on Luria-Bertani broth (1% (*w*/*v*) NaCl, 1% (*w*/*v*) Tryptone, 0.5% (*w*/*v*) Yeast Extract, pH 7.0) plates supplemented with kanamycin, while the bottom was LB plates without kanamycin. (**B**) Plasmid yields of TtAgo expression vector in BL21 (DE3) at different times (2, 3, and 4 h) post-IPTG induction and before induction (0 h). Means and standard deviations from three biological replicates, error bars represent the mean ± s.d. (**C**) Colony-forming units were determined after plasmids recovered from before induction (0 h), different time points (2, 3, and, 4 h) of post-IPTG induction and without induction (the last column labeled as 4* represents 4 h of growth without IPTG induction) were re-transformed into *E. coli* BL21 (DE3) (means and standard deviations from three independent biological replicates, *p* < 0.05, *p* < 0.01, *** *p* < 0.001) (**D**) Spot plating assay for the effect of NgAgo expression on host cell genome and its expression vector degradation. (**E**) Plasmid yields of *Natronobacterium gregoryi* (NgAgo) expression vector in BL21 (DE3) at different times (2, 3, and 4 h) post-IPTG induction and before induction (0 h) as noted in (**B**). (**F**) Colony-forming units were determined after plasmids recovered from before induction (0 h), different time points of post-IPTG induction and without induction as noted in (**C**). (**G**) Spot plating assay for the effect of RsAgo expression on host cell genome and its expression vector degradation. (**H**) Plasmid yields of *Rhodobacter sphaeroides* (RsAgo) expression vector in BL21 (DE3) at different times (2, 3, and 4 h) post-IPTG induction and before induction (0 h) as noted in (**B**). (**I**) Colony-forming units were determined after plasmids recovered from before induction (0 h), different time points of post-IPTG induction and without induction as noted in (**C**).

**Figure 2:**
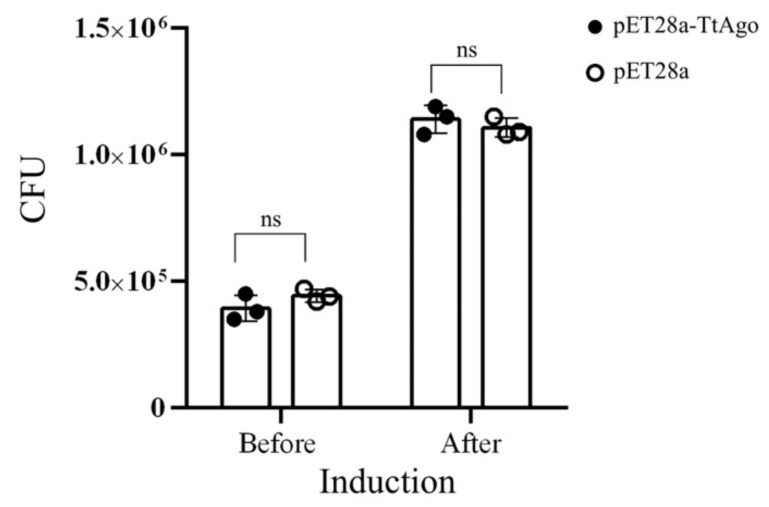
TtAgo induction has no effect on bacterial growth. Means and standard deviations from three biological replicates, error bars represent the mean ± s.d. “ns” represents “not significant”. *p* < 0.05, *p* < 0.01, *p* < 0.001.

As shown by spot plating assays (Figure 1A), the transformed cells grew well on LB medium supplemented with kanamycin without IPTG induction; however, after the addition of IPTG into the medium, no colonies formed on plates with kanamycin selection. Thus, TtAgo expression inhibited cell proliferation under plasmid selection pressure with kanamycin. To further investigate how TtAgo expression suppressed colony formation, we plated the transformed cells without kanamycin. As shown in Figure 1A, the transformed cells with IPTG induction grew as well on LB plate without kanamycin as uninduced cells. Under the conditions of TtAgo expression and in the absence of kanamycin selection, there is no difference in CFU analysis between pET28a-TtAgo and pET28a, before and after IPTG induction (Figure 2). These results suggested that the expression of TtAgo protein is not terminally toxic to the BL21 (DE3) host cells and thus does not permanently damage the *E. coli* genome.

We next investigated whether TtAgo expression might cause a decrease in the amount or copy number of its expression vector by examining plasmid yields isolated from the transformed cells at different times post-IPTG induction. As shown in Figure 1B,C, plasmid yields decreased as IPTG induction time increased, and plasmid recovered from cells induced with IPTG yielded fewer colonies. This suggested that induction of TtAgo expression decreased the relative levels of its expression vector.

**Figure 3:**
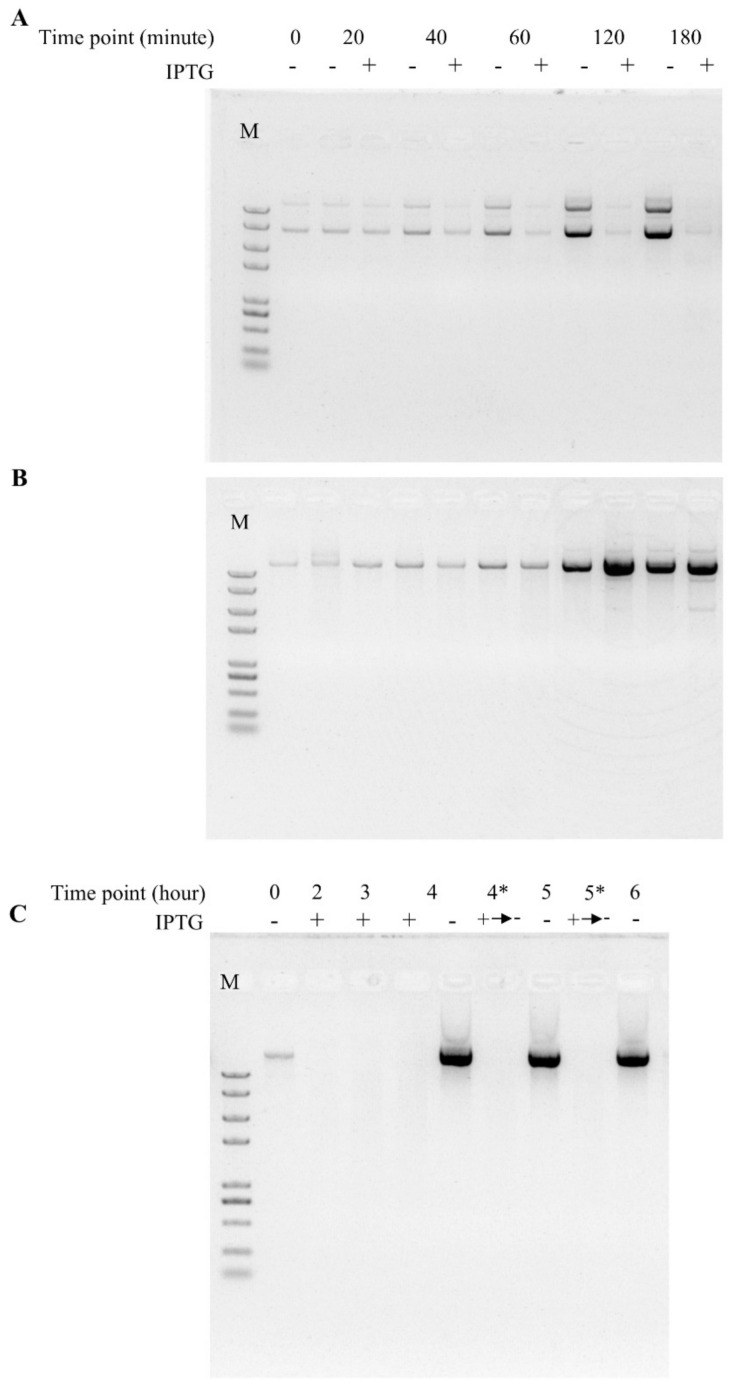
TtAgo induction reduces recovery of the expression vector. pET28a-TtAgo and pET28a plasmids were extracted from 1 mL of cell culture under inducing or non-inducing conditions and analyzed by agarose gel electrophoresis. (**A**) Equal volume of extracted pET28a-TtAgo plasmid was loaded. “Time point” represents the bacterial growth time after OD_600_ reaches 0.5, and IPTG induction and no induction treatments were indicated as “+” and “−”. M, 2K Plus II marker (TransGen), suitable for all three figures. (**B**) Equal volume of extracted pET28a plasmid was loaded. “Time point” represents the bacterial growth time after OD_600_ reaches 0.5, and IPTG induction and no induction treatments were indicated as “+” and “−”. (**C**) Equal volume of extracted pET28a-TtAgo plasmid was loaded. “Time point” represents the bacterial growth time after OD_600_ reaches 0.5, and IPTG induction and no induction treatments were indicated as “+” and “−”. “4*” represents 4-h IPTG induction growth plus another 1-h no induction growth, consistent with the “+→−” label on IPTG induction treatment; “5*” represents 4-h IPTG induction growth plus another 2 h no induction growth, consistent with the “+→−” label on IPTG induction treatment.

We then asked whether this activity was unique to TtAgo or a common feature to other pAgos by repeating these experiments with two other prokaryotic argonaute genes, RsAgo and NgAgo. Similar to TtAgo, both RsAgo and NgAgo similarly blocked colony formation on selective medium post induction (Figure 1D–I). These observations demonstrated that three different pAgo proteins expressed from pET28a expression vector in E. coli BL21(DE3) host cells at 37 °C function similarly after IPTG induction to reduce apparent growth. By examining the plasmids on agarose gel from different times post-IPTG induction, we demonstrated that the pET28a-TtAgo plasmid yields decreased as IPTG induction time increased, and in contrast, the plasmid yields from no IPTG induction groups increased as growth time increased (Figure 3A). In contrast, the plasmid yields of the control plasmid pET28a increased as the IPTG induction time was extended (Figure 3B). Furthermore, after removing the IPTG and allowing an extra 2h growth, we did not observe a recovery of plasmid levels, strongly suggesting that TtAgo expression resulted in irreversible plasmid loss (Figure 3C).

**Figure 4:**
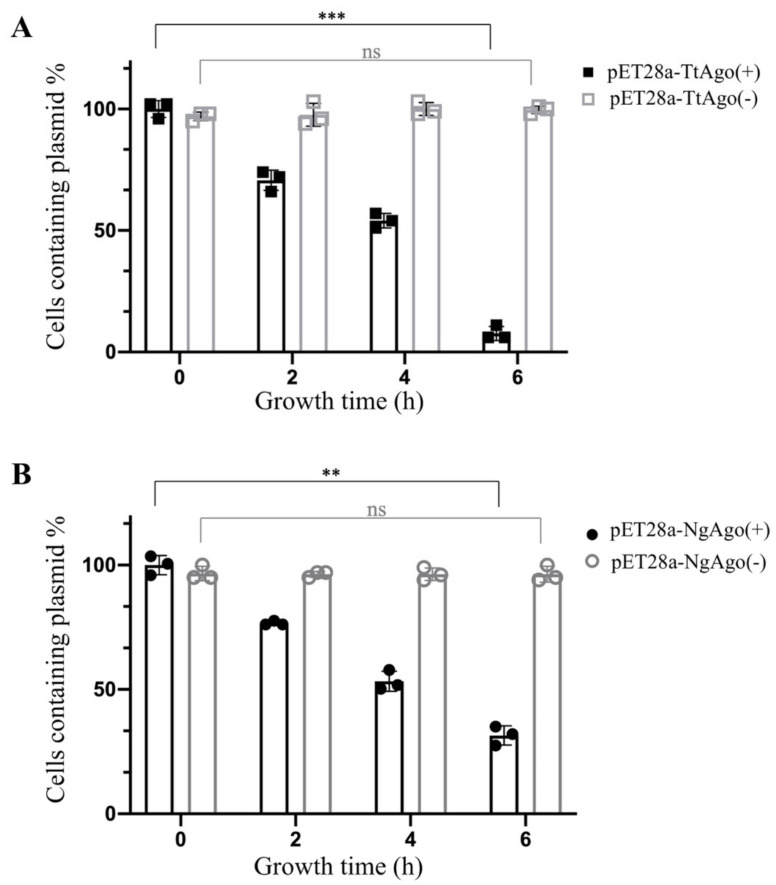
Plasmid maintenance assay of pET28a-TtAgo and pET28a-NgAgo with and without IPTG induction. (**A**) Cells containing pET28a-TtAgo plasmid were calculated before and after different times (2, 4, and 6 h) post-IPTG induction, and without IPTG induction. Legend “pET28a-TtAgo(+)” represents IPTG induction treatment, and legend “pET28a-TtAgo(−)” represents no-induction treatment. The normalization of data was performed with paired-samples *t* test (means and standard deviations from three independent biological replicates, error bars represent the mean ± s.d. ** *p* < 0.01, *** *p* < 0.001, “ns” represents “not significant”.). (**B**) Cells containing pET28a-NgAgo plasmid were calculated before and after different times (2, 4, and 6 h) post-IPTG induction, and without IPTG induction. Legend “pET28a-NgAgo(+)” represents IPTG induction treatment, and legend “pET28a-NgAgo(−)” represents no-induction treatment. The normalization of data was performed with paired-samples *t* test (means and standard deviations from three independent biological replicates, error bars represent the mean ± s.d., ** *p* < 0.01, “ns” represents “not significant”. *** *p* < 0.001).

To further verify the effect of pAgo proteins on their expression plasmids, we carried out a plasmid maintenance assay. We picked three individual colonies from the pET28a-TtAgo and pET28a-NgAgo transformation plates and inoculated them into 10 mL LB+Kan liquid medium and cultured at 37 °C until OD600 reached 0.5. Five milliliters of the cell culture in each transformation group were dispensed into four tubes with 1 mL each. The first tube was immediately stored at 4 °C refrigerator used as the control, while the remaining 3 tubes were allowed to grow at 37 °C with IPTG induction for 2, 4, and 6h. Each sample was then examined for plasmid maintenance efficiency by plating the samples on both LB and LB+Kan plates. The other 5 mL of culture for each transformation group was treated in the same way, except that no IPTG inducer was added. As shown in Figure 4, the plasmid-containing cells significantly decreased as the IPTG induction time increased, while there was no difference in the no induction treatment group, both for pET28a-TtAgo and pET28a-NgAgo. These results suggested that the expression of pAgo proteins resulted in the loss of plasmids.

### 3.2. Effect of Deletion Mutant TtAgo (ΔTtAgo) Protein on Cell Growth and Plasmid Yields

**Figure 5:**
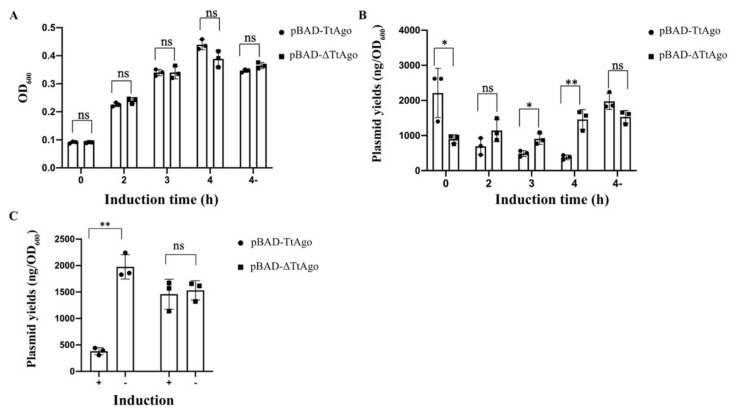
The effect of TtAgo and ΔTtAgo expression on bacterial cell growth and plasmid yields. (**A**) OD_600_ of pET28a-pBAD-TtAgo and pET28a-pBAD-ΔTtAgo transformed cell culture was measured before and after different times (2, 3, and 4 h) post-arabinose induction or 4 h growth without arabinose (labeled as “4-”). The data were used to perform independent-samples *t* test (means and standard deviations from three independent biological replicates, error bars represent the mean ± s.d. * *p* < 0.05, ** *p* < 0.01, “ns” represents “not significant”.). (**B**) pET28a-pBAD-TtAgo and pET28a-pBAD-ΔTtAgo plasmids were extracted before and after different times (2, 3, and 4 h) post-arabinose induction and4 h growth without arabinose (labeled as “4-”). The data were used to perform independent-samples *t* test (means and standard deviations from three independent biological replicates, error bars represent the mean ± s.d. * *p* < 0.05, ** *p* < 0.01, “ns” represents “not significant”.). (**C**) Plasmid yields of pET28a-pBAD-TtAgo and pET28a-pBAD-ΔTtAgo from 4-h induction (labeled as “+”) and no-induction treatment (labeled as “−”) were used to perform paired-samples *t* test (means and standard deviations from three independent biological replicates, error bars represent the mean ± s.d. * *p* < 0.05, ** *p* < 0.01, “ns” represents “not significant”.).

To further confirm that the plasmid loss was due to functional pAgo expression, we then generated a TtAgo deletion mutant (ΔTtAgo) by deleting an internal 573 bp sequence, removing amino acids (aa) 25 to 215. The mutant construct was designated as pET28a-pBAD-ΔTtAgo and used to transform E. coli BL21(DE3) competent cells. Three colonies from each transformation group were picked and inoculated into 4 mL LB+Kan liquid medium. Cell cultures were subdivided into two tubes when OD600 reached 0.5. One was treated with arabinose for induction of pAgo gene expression and the OD600 was measured at 2, 3, and 4 h post induction; the other tube was continuously cultured without arabinose for 4 h and OD600 was measured. TtAgo-transformed cells grew as well as those transformed with a vector expressing the presumably defective TtAgo mutant with a large deletion, regardless of arabinose induction (Figure 5A). In contrast, the plasmid yields difference between two vector groups was significant after 4 h of induction (Figure 5B). Four-hour arabinose induction treatment caused a significant decrease of plasmid yields in the wild-type TtAgo vector group, but not for ΔTtAgo (Figure 5C). These data provided further evidence that the expression of functional TtAgo protein caused the loss of TtAgo-encoded plasmid, while no effect on host cell growth was observed.

### 3.3. In Vivo Activity Comparison Between TtAgo Wild Type and 3′ Terminus Modification

**Figure 6:**
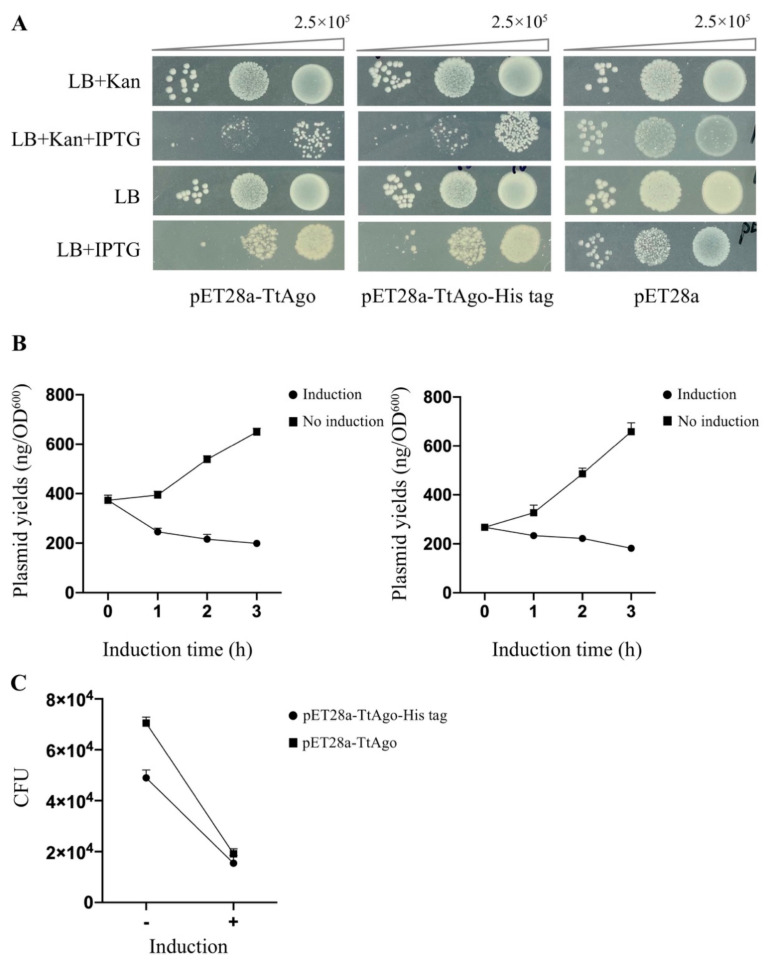
Activity comparison between TtAgo wild type and its 3′ terminus modification. (**A**) Spot plating assay for the comparison of pET28a-TtAgo, pET28a-TtAgo-His tag, and pET28a effects on the host cell genome and their expression plasmid yields. Cells were grown with or without IPTG as noted. The upper panels were grown on LB plates supplemented with kanamycin, while the bottom was LB plates without kanamycin. (**B**) Plasmid yields of TtAgo expression vector (pET28a-TtAgo, left vs. pET28a-TtAgo-His tag, right) in BL21 (DE3) at different times post-IPTG induction (means and standard deviations from three biological replicates, error bars represent the mean ± s.d.). (**C**) Colony-forming units were determined after the plasmids recovered from 2-h IPTG induction treatment and no induction treatment were re-transformed into *E. coli* BL21 (DE3).

To leverage the discoveries obtained above for further function analysis of TtAgo in vivo, we attempted to establish a high-throughput screening system for isolating a loss-of-function of TtAgo point mutations. To this end, we compared wild-type TtAgo with a construct with a C-terminal modification. We fused a 6 × His tag at the 3′ end of TtAgo coding sequence in pET28a-TtAgo vector (pET28a-TtAgo-His tag). By spot plating assays (Figure 6A), measuring plasmid yields (Figure 6B) and CFU assay (Figure 6C) in parallel with pET28a-TtAgo vector, we found that a C-terminal modification of TtAgo did not abolish its activity in E. coli, with only a slight decrease in its activity when compared with pET28a-TtAgo in the CFU assay. This observation laid the foundation for establishing a high-throughput screening system with a TtAgo fused to a marker gene.

### 3.4. Establishment of a High Throughput Screening System to Isolate Loss-of-Function Point Mutations of TtAgo in Vivo

To further characterize the ability of TtAgo to cause the loss of the plasmids, we devised a selection system to identify loss-of-function point mutations in TtAgo. We employed error-prone PCR (EP-PCR)-mediated random mutagenesis in a high-throughput screening system to isolate loss-of-function mutants of TtAgo. The disadvantage of EP-PCR-mediated mutagenesis is often the introduction of a stop codon, which confers a loss-of-function phenotype that is relatively uninformative. To eliminate stop-codon-containing mutants from our screen, we adopted a marker gene fusion strategy, i.e., fusing a marker gene downstream of the TtAgo gene. Initial experiments fusing the ampicillin resistance gene downstream of *TtAgo* in the pET28a expression vector backbone resulted in unacceptable background levels after large-scale ligation of the EP-PCR product into the expression vector and electroporation. Plasmids recovered from this approach reflected contamination, which could not be eliminated despite substantial effort. We next focused on the *E. coli fabI* gene, as it has been reported that overexpression of *fabI* confers resistance to triclosan, an FDA-approved non-antibiotic biocide [36,37]. We incorporated this mutant *fabI* gene (*fabI* G93V) [40] into our screening system by fusing it in-frame downstream of *TtAgo*, allowing expression of a TtAgo-FabI fusion protein.

**Figure 7:**
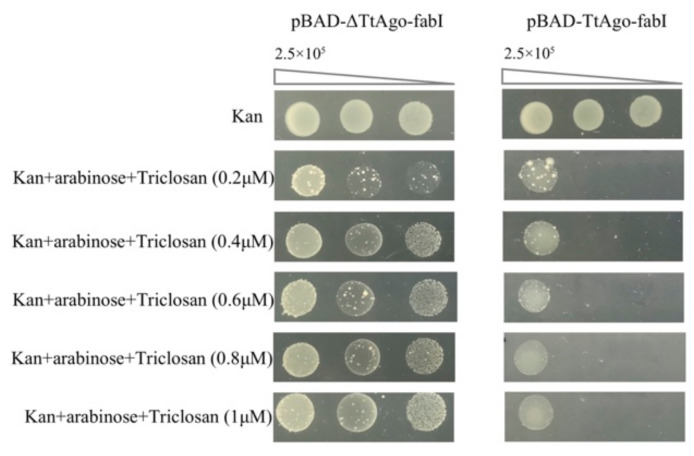
Spot plating assay for optimizing concentration of chemical triclosan. pET28a-ΔTtAgo-fabI plasmid encoding a truncated TtAgo with a large in-frame deletion was used as a loss-of-function control for enzymatic dead TtAgo activity. Two biological replicates each for pET28a-pBAD-ΔTtAgo-fabI and pET28a- pBAD-TtAgo-fabI were performed for each concentration.

Compared with antibiotic resistance genes, fabI has not been widely used in plasmid construction. Thus, incorporation of fabI gene into our high-throughput screening system could potentially overcome the plasmid contamination issue. As a proof of concept, and to optimize the system, we constructed both a wild-type and a deletion mutant of TtAgo fused with fabI and tested them for effect on E. coli growth with different concentrations of triclosan. ΔTtAgo is predicted to exhibit a loss-of-function phenotype as a consequence of its large deletion. After transformation with wild-type and ΔTtAgo expression vectors and drug selection, three random, independent colonies in each group were picked and expanded in LB for 4 h. These cultures were subjected to the spot plating assay on LB plates supplemented with kanamycin, L-arabinose, and different concentrations of triclosan. As shown in Figure 7, the optimal concentration of triclosan was determined to be 1μM. With this optimized triclosan concentration, expression of wild-type TtAgo fused with fabI yielded no visible colonies in diluted spots. In contrast, E. coli expressing ΔTtAgo fused to fabI could survive the triclosan selection. This demonstrated that the TtAgo-FabI fusion retains the wild-type TtAgo function as observed above.

### 3.5. Construction of a Random Mutation Library of TtAgo by EP-PCR

Using optimized EP-PCR conditions, we carried out large-scale EP-PCR reactions to amplify the coding region of *TtAgo*. After purification, PCR products were ligated into the expression vector pET28a-pBAD-TtAgo-fabI. Induction of TtAgo from this vector with L-arabinose was more tightly controlled that that mediated by IPTG with the T7 promoter in the previous systems. These vectors were purified and used to electroporate *E. coli* BL21(DE3) competent cells. Colonies were recovered from LB selection plates supplemented with kanamycin, triclosan, and L-arabinose and were sent for Sanger sequencing. Under our optimized conditions, we achieved 5 × 10^5^/ng transformation efficiency, and we transformed about 100 ng of ligation product for TtAgo library screening. All colonies were expanded, and plasmids were recovered. Recovered plasmids were re-transformed into BL21 (DE3) cells for phenotype confirmation and 42 confirmed clones were sequenced. The sequencing results are summarized in Table 3. Most mutants contained multiple point mutations, with some mutations appearing in multiple independent mutants. Synonymous mutations such as G388G in mutant number 11 or G186G, L409L in mutant number 12 were excluded from Table 3 and were not studied further since the amino acid was not changed.

Some point mutations were re-constructed by site-specific mutagenesis and verified one by one for phenotype. Many of these mutations showed similar activity to wild-type TtAgo. For example, in the mutant 1 in Table 3, P97S, G150R, and L189Q were re-constructed, separately, but only G150R showed a loss-of-function phenotype. In addition, some mutants, such as mutant 5, 6, and 7 listed in Table 3, still retained wild-type activity after induction, indicating false positive cases. Ultimately, eight point mutations were finally confirmed to demonstrate loss-of-function phenotype (summarized in Table 1).

### 3.6. Function Analysis of TtAgo Point Mutations

**Figure 8:**
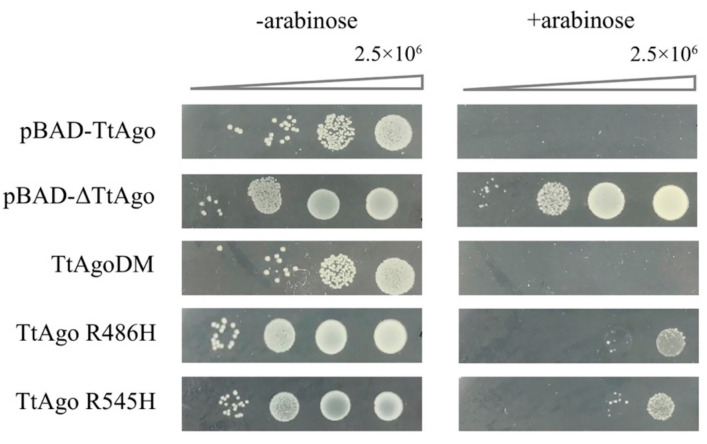
Colony spot plating assay for TtAgo point mutations. Colony spot plating assay for pET28a-pBAD-TtAgo, pET28a-pBAD-ΔTtAgo, pET28a-pBAD-TtAgoDM, pET28a-pBAD-TtAgo (R486H), pET28a-pBAD-TtAgo (R545H), with and without L-arabinose induction. pET28a-pBAD-TtAgo represents intact TtAgo enzyme activity, and pET28a-pBAD-ΔTtAgo represents enzymatic dead TtAgo activity. Three biological replicates were performed for each group. “-arabinose” represents without arabinose induction, “+arabinose” represents with arabinose induction.

The TtAgo protein, like other argonaute proteins, has four domains. These include the N-terminal domain (aa5-96), PAZ domain (aa186-276), MID domain (aa336-442), and PIWI-like domain (aa442-667) [47] (Figure S4). The PIWI-like domain is thought to possess the catalytic ability to degrade target DNA or RNA due to the conserved tetrad catalytic center [20]. All of the currently described dead TtAgo mutations are located in the PIWI-like domain, such as the two symmetrical arginine residues (R545, involved in the cleavage reaction, and R486, not involved in target cleavage), which play distinct roles in ssDNA target cleavage [48], and TtAgoDM (Double mutant; D478A, D546A), which showed no cleavage activity in in vitro cleavage assay [31]. Surprisingly, no mutations within the PIWI domain were isolated in our high-throughput screen. As controls for our experiments, we reconstructed three of these previously studied mutants in the literature [31,48]. These included TtAgoDM (D478A, D546A) and single mutants in the two symmetrical arginine mutations (R486H and R545H). However, after cloning these mutants in the pET28a-pBAD-TtAgo expression vector, they behaved like wild-type TtAgo protein in the spot plating assay (Figure 8).

**Figure 9:**
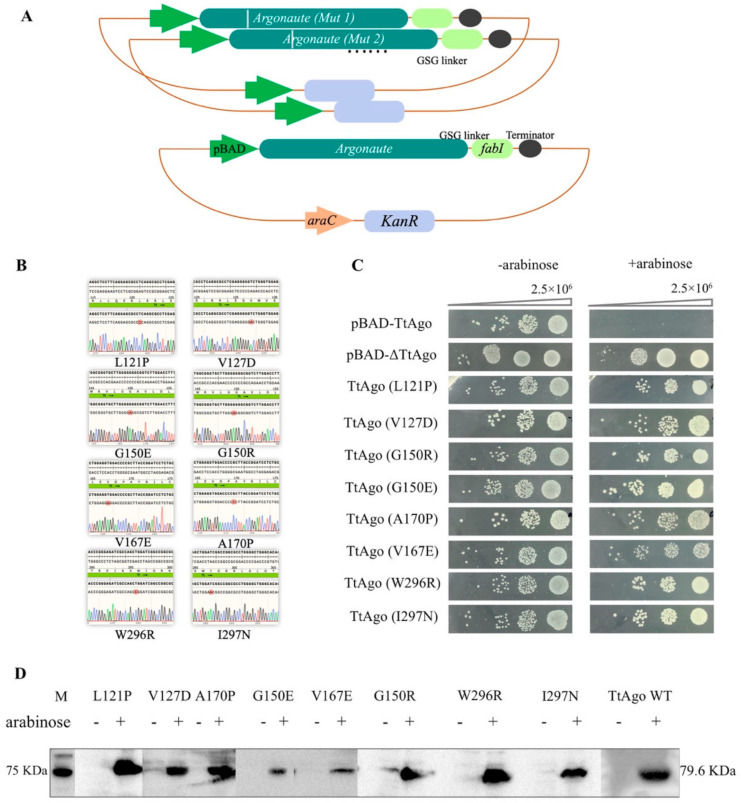
Function analysis of eight isolated loss-of-function TtAgo single point mutations in BL21(DE3). (**A**) Schematic diagram of the construction of random mutant library in pET28a-pBAD-TtAgo-fabI vector. Mutations introduced by error-prone PCR (EP-PCR) are indicated as grey bars in the TtAgo coding region. (**B**) Sequencing results from the eight isolated TtAgo loss-of-function mutants. Mutated nucleotide is shown in red. (**C**) Colony spot plating assay for pET28a-pBAD-TtAgo, pET28a-pBAD-ΔTtAgo, and eight isolated TtAgo loss-of-function mutant, with and without L-arabinose induction. The pET28a-pBAD-TtAgo represents intact TtAgo enzyme activity, and pET28a-pBAD-ΔTtAgo represents enzymatic dead TtAgo activity. Three biological replicates were performed for each group. “-arabinose” represents without arabinose induction, “+arabinose” represents with arabinose induction. (**D**) Protein expression examination by Western blotting. The *E. coli* BL21 (DE3) competent cells were transformed with each of the eight re-constructed pET28a-pBAD-mTtAgo plasmids, and pET28a-pBAD-TtAgo plasmid. Protein expression was induced with L-arabinose (10 mM) for 4 h when OD _600_ reached approximately 0.5. Total protein was extracted for Western blotting with His-Tag Mouse Monoclonal antibody.

In addition to the conserved domains described above, there are two linker regions in TtAgo, Linker 1 (aa96-186) and Linker 2 (aa276-336), which connect the four domains. Interestingly, we identified eight single point mutations, of which six (TtAgo L121P, TtAgo V127D, TtAgo G150R, TtAgo G150E, TtAgo V167E, and TtAgo A170P) were in the Linker 1 domain and two (TtAgo W296R, TtAgo I297N) were in the Linker 2 domain. To verify the effect of the eight individual mutations in the linker regions identified by the loss-of-function screen, we reconstructed them de novo by site directed mutagenesis (Figure 9A,B). All of these single-point mutants were retransformed into BL21(DE3) competent cells and subjected to the spot assays on LB plates supplemented with kanamycin, with and without L-arabinose. All eight mutants exhibited loss-of-function in the spot assays (Figure 9C).

To rule out the possibility that the loss-of-function mutation phenotype was due to decreased expression of the mutant proteins, we examined their expression by Western blot. As shown in Figure 9, all mutant proteins were expressed (Figure 9D), suggesting that the loss-of-function phenotype was not simply due to a loss of expression.

**Figure 10:**
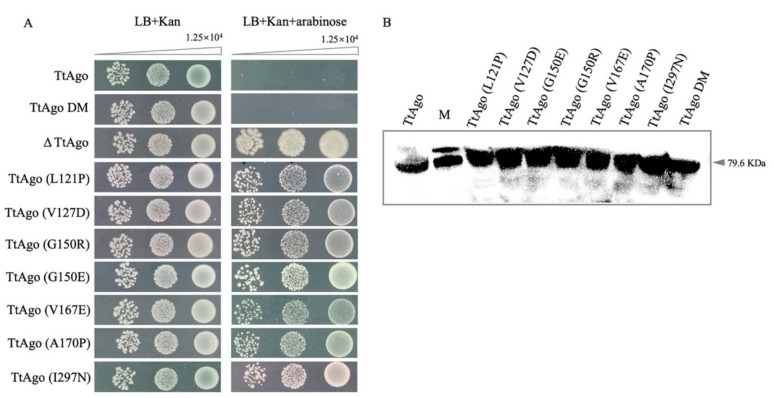
Function analysis of TtAgo mutants in *E. coli* DH5α. (**A**) Colony spot plating assay for pET28a-pBAD-TtAgo, pET28a-pBAD-TtAgoDM, pET28a-pBAD-ΔTtAgo, and randomly picked TtAgo mutants, with and without L-arabinose induction. The pET28a-pBAD-TtAgo represents intact TtAgo enzyme activity, and pET28a-pBAD-ΔTtAgo represents enzymatic dead TtAgo activity. Three biological replicates were performed for each group. (**B**) Protein expression examination by Western blotting. Cell cultures from (A) were inoculated into 4 mL LB+Kan medium and were induced with L-arabinose (10 mM) for 4 h when OD_600_ reached approximately 0.5. Total protein was extracted for Western blotting with His-Tag Mouse Monoclonal antibody.

To eliminate the possibility that the loss-of-function mutants represent an artefact of the *E. coli* system. The effect of the mutations has been tested in *E. coli* DH5α, a homologous system to BL21(DE3). Similar spot assay results were obtained from DH5α (Figure 10A) and the protein was successfully and effectively expressed after 4 h of arabinose induction (Figure 10B). These results confirmed that these mutants isolated by FabI-Triclosan selection are functionally deficient.

### 3.7. Structural Simulation of Isolated TtAgo Point Mutations

**Figure 11:**
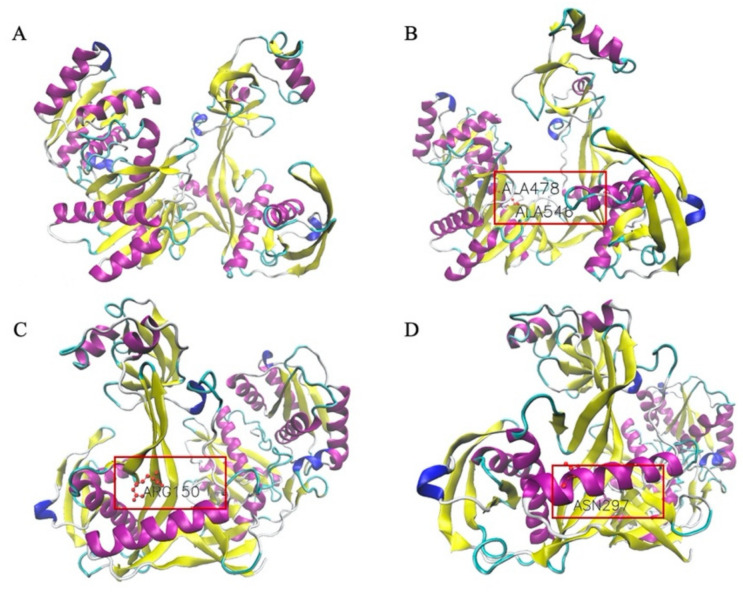
Structural simulation of TtAgo, TtAgoDM, TtAgo (G150R), and TtAgo (I297N). The figures were drawn in newcartoon method and colored by secondary structure, with alpha helix indicated in purple, beta-sheet in yellow, and amino acids colored in CPK (ball-and-stick model) style. (**A**) Structural simulation of TtAgo protein in apo form. (**B**) Structural simulation of TtAgo DM protein. (**C**) Structural simulation of TtAgo (G150R) protein. (**D**) Structural simulation of TtAgo (I297N) protein.

The effect of the eight individual loss of function mutations on overall TtAgo structure was simulated in silico. None altered the predicted structure of the TtAgo protein, and the protein structure simulation diagrams of TtAgo (Figure 11A), TtAgo DM (Figure 11B), TtAgo (G150R) (Figure 11C), and TtAgo (I297N) (Figure 11D) are shown in Figure 11, with and the location of the mutated amino acids framed in red boxes. The protein structure simulations of the rest of TtAgo mutants are included in Figure S7 in the Supplementary Information.

## 4. Discussion

In general, pAgos are thought to play a role in host defense by interfering with invading nucleic acids. However, studies of their mechanism of action in their natural host cells remain elusive. The extreme thermophile *Thermus thermophilus* is a gram-negative aerobic bacterium with optimal growth temperature at 65 °C. The pAgo of *Thermus thermophilus* (TtAgo) has been reported to use small, single-stranded DNA as a guide to mediate host defense by targeting invading DNA complementary to the guide [4]. The requirement of high temperatures for *Thermus thermophilus* growth makes it difficult to characterize TtAgo in its host cells. This lack of a laboratory model has hindered our understanding of the mechanism by which TtAgo defends its natural host cells against invasive DNA, whether plasmid [12] or phage in nature. In the present study, we expressed TtAgo in *E. coli* BL21 (DE3) cells at 37 °C and determined that expression of TtAgo recombinant protein strongly decrease the levels of its expression vector. Compared with the control pET28a plasmid or vector expressing deficient ΔTtAgo, the failure to detect high levels of recombinant TtAgo expression at 37 °C was specifically due to effects on its own expression plasmid. This occurred without inhibiting bacterial growth, which is reminiscent of TtAgo behavior in its natural host, *T. thermophilus* [12].

We further explored this phenomenon of TtAgo causing loss of its expression plasmid as an in vivo model of function in heterologous *E. coli* BL21 (DE3) cells, and successfully established a high throughput screening system to select loss-of-function mutants of TtAgo in vivo. Eight single amino acid changes identified in these loss-of-function mutants of the TtAgo protein were validated after de novo reconstruction both in BL21(DE3) and DH5α.

Somewhat surprisingly, analysis of the loss-of-function mutants revealed that all were located in the L1 and L2 linker regions between the four conserved structural domains. These linkers (L1 and L2) connect the two lobes, as revealed by structural studies of pAgos [47]. The linkers also play a role in target recognition and binding. Specifically, Zhu et al. 2016 [49] identified a flexible hinge between linker 1 and the PAZ domain, which may play a role in guide DNA loading. Klum et al. 2017 [50] also proposed that helix-7 in linker 2 of hAgo2 acts as a catalyst for seed-pairing, accelerating target binding and dissociation, and upon recognition of a matching target strand, helix-7 in linker 2 moves and stabilizes base pairing by interactions with the minor groove of the guide-target duplex. Elad et al. (2012) also found that although miRNA interact with all four domains of hAgo2, they also interact with the two linkers. Additionally, an isoleucine that protrudes from linker 2 was involved in kinking bases 6 and 7 of the miRNA seed region [51]. Similarly, Doxzen et al. (2017) reported that Linker 2 domain of MpAgo interacts with the phosphate backbone to stabilize the contorted 3′ end of the DNA target [25]. Overall, it is believed that the linker helix plays an important role in the formation of the binary, as well as the ternary complex [26]. Studies of TtAgo in *P. multocida* (with an optimal growth temperature at 35 °C ± 5 °C) demonstrated that it can enhance homologous sequence-directed recombination [6], demonstrating that TtAgo can play other roles in bacterial cells grown below their natural growth temperature. Our data strongly suggested that the linker regions of TtAgo play a crucial role in causing its expression plasmid loss in vivo.

Interestingly, our screen did not isolate any mutations in the catalytic center, possibly because that type of mutant may still bind to the plasmid locked as a frozen intermediate and that can still cause the loss of the plasmid. However, based on the observation that TtAgoDM (Double mutant; D478A, D546A) demonstrated similar activity to wild-type TtAgo in the spot assay, we suggested that TtAgo utilizes a different functional mechanism to induce plasmid loss from the one observed in vitro. Future studies are warranted to more clearly elucidate the mechanisms responsible for TtAgo in vivo specifically causing its expression plasmid loss.

## 5. Conclusions

By comparing plasmid content and cell density among three different pAgos (TtAgo, NgAgo, and RsAgo), mutant TtAgo and control vector transformed cells, we concluded that TtAgo expression specifically leads to its expression vector loss in *E. coli* BL21(DE3), without harming bacterial genomic DNA. By employing the *fabI* gene as a novel selection marker, eight TtAgo single point mutants were identified that conferred loss of function. Six mutations were located in the Linker 1 domain and two were in the Linker 2 domain. These findings provide novel information about TtAgo function and establish a system to study the molecular basis of this activity.

## Figures and Tables

**Table 1:** Eight isolated single point mutations of *Thermus thermophilus* prokaryotic argonaute (TtAgo) loss-of-function mutants.

Mutation	Location
L121P	Linker 1
V127D	Linker 1
G150R G150E V167E A170P W296R I297N	Linker 1 Linker 1 Linker 1 Linker 1 Linker 2 Linker 2

**Table 2:** Plasmids used and presented in Figures 8 and 9.

Plasmid Name	Description
pET28a-pBAD-TtAgo (R486H)	Arginine 486 replaced with histidine in TtAgo
pET28a-pBAD-TtAgo (R545H)	Arginine 545 replaced with histidine in TtAgo
pET28a-pBAD-TtAgoDM (D478A, D546A)	Aspartic acid 478 and 546 replaced with alanine in TtAgo
pET28a-pBAD-ΔTtAgo	Amino acids 25 to 215 of TtAgo deleted
pET28a-pBAD-TtAgo (L121P)	Leucine 121 replaced with proline in TtAgo
pET28a-pBAD-TtAgo (V127D)	Valine 127 replaced with aspartic acid in TtAgo
pET28a-pBAD-TtAgo (G150R)	Glycine 150 replaced with arginine in TtAgo
pET28a-pBAD-TtAgo (G150E)	Glycine 150 replaced with glutamic acid in TtAgo
pET28a-pBAD-TtAgo (V167E)	Valine 167 replaced with glutamic acid in TtAgo
pET28a-pBAD-TtAgo (A170P)	Alanine 170 replaced with proline in TtAgo
pET28a-pBAD-TtAgo (W296R)	Tryptophan 296 replaced with arginine in TtAgo
pET28a-pBAD-TtAgo (I297N)	Isoleucine 297 replaced with asparagine in TtAgo

**Table 3:** Sequencing result of screened mutant of TtAgo.

Colony Number	Mutation Position and Type
1	P97S, G150R, L189Q
2	V127D
3 4 5 6 7 8 9 10 11 12 13 14 15 16, 40 17 18, 21, 22 19 20 23 24 25 26, 28 27, 30 29 31 32 33 34 35, 41 36 37 38 39 42	A278T, W296R V127D, R651W A555V, E483D A555V, A659S A555V, L556P G150R A53E, R59H, G144R R427M, E483D A133V, V127D A151E, I297N L265P, A278T, W296R V127D, R286Q, R299Q G150E, L430H G150E P97S, G150R, L189Q, E210K, H637Y L265P, A278T, W296R V127D, R651W A113V, V127D, G388G F10Y, R123H, G150E, G220S, L638Q P37L, V127D, A133V V167E G150W, G467D L121P L174P, I293F, L430P, K599Q A170P E41A, G126E, R136Q, R141Q, A345T G150E A114V, V127D, A245V, F485L L75F, I293F R122G, G150R, S229P, R427S R155K, G150W, A555P G126E, G150E L148P L121P, R420H

## Data Availability

Not applicable.

## Supplementary Materials

The following are available online at https://www.mdpi.com/article/10.3390/biom11040524/s1, Figure S1: Full-length agarose gel electrophoresis result of pET28a-TtAgo plasmid with different treatments; Figure S2: Full-length agarose gel electrophoresis result of pET28a plasmid with different treatments; Figure S3: Full-length agarose gel electrophoresis result of pET28a-TtAgo plasmid with different treatments; Figure S4: Linear representation of the 4 domains and 2 linkers of the TtAgo protein; Figure S5: Full-length western blotting images of TtAgo and TtAgo mutant protein expression in BL21(DE3); Figure S6: Full-length western blotting image of TtAgo and TtAgo mutant protein expression in DH5α; Figure S7: Protein structural simulation figures of the rest of the TtAgo mutants; Table S1: Primers used in this experiment; Table S2: Error-prone PCR reaction system.
