# Supplementary material for: Expression and Functional Analysis of the Argonaute Protein of Thermus thermophilus (TtAgo) in E. coli BL21(DE3)
Source: Biomolecules. 2021 Mar 31;11(4):524. doi: 10.3390/biom11040524 (PMC8067300; doi:10.3390/biom11040524)
Supplement: Supplementary file 1 [file biomolecules-11-00524-s001.pdf]

Supplementary Information

Expression and functional analysis of the argonaute protein of *Thermus thermophilus* (TtAgo) in *E. coli* BL21(DE3)

Jiani Xing, Lixia Ma, Xinzhen Cheng, Jinrong Ma, Ruyu Wang, Kun Xu, Joe S. Mymryk and Zhiying Zhang

Inventory of Supplemental Information

Figure S1. Full-length agarose gel electrophoresis result of pET28a-TtAgo plasmid with different treatments, related to Figure 3A in the revised manuscript.

Figure S2. Full-length agarose gel electrophoresis result of pET28a plasmid with different treatments, related to Figure 3B in the revised manuscript.

Figure S3. Full-length agarose gel electrophoresis result of pET28a-TtAgo plasmid with different treatments, related to Figure 3C in the revised manuscript.

Figure S4. Linear representation of the 4 domains and 2 linkers of the TtAgo protein.

Figure S5. Full-length western blotting images of TtAgo and TtAgo mutant protein expression in BL21(DE3), related to Figure 9D in the revised manuscript.

Figure S6. Full-length western blotting image of TtAgo and TtAgo mutant protein expression in DH5α, related to Figure 10B in the revised manuscript.

Figure S7. Protein structural simulation figures of the rest of the TtAgo mutants.

Table S1. Primers used in this experiment

Table S2. Error-prone PCR reaction system

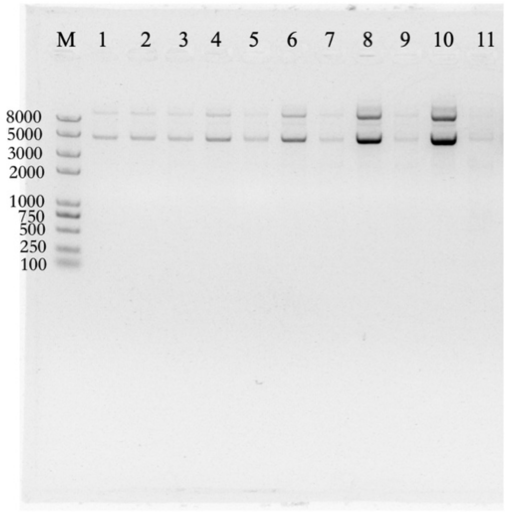

Figure S1. Full-length agarose gel electrophoresis result of pET28a-TtAgo plasmid with different treatments, related to Figure 3A in the revised manuscript. Agarose gel was imaged with Gel Imager (MicroChemi, DNR) with 100 ms exposure time. Photoshop was used to add the label of molecular size markers and lane number.

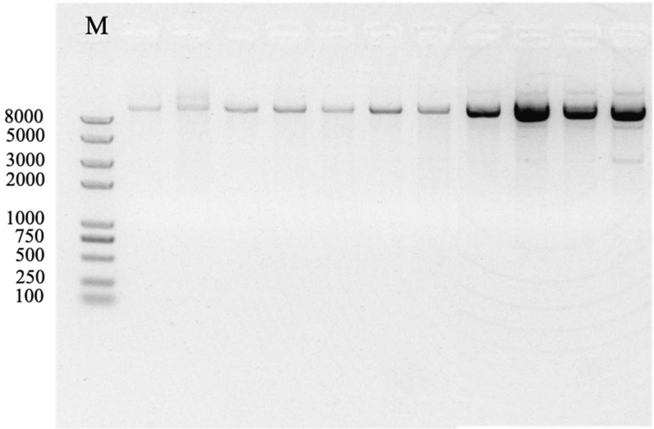

Figure S2. Full-length agarose gel electrophoresis result of pET28a plasmid with different treatments, related to Figure 3B in the revised manuscript. Agarose gel was imaged with Gel Imager (MicroChemi, DNR) with 100 ms exposure time. Photoshop was used to add the label of molecular size markers marker.

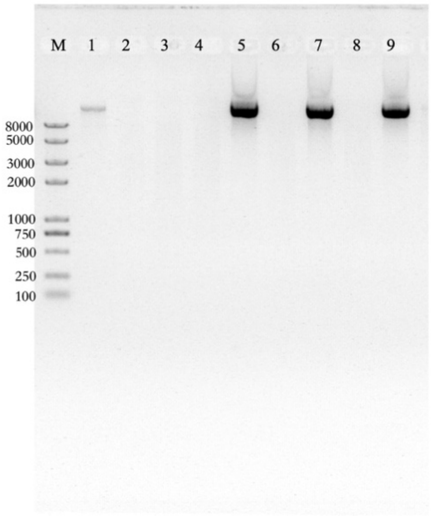

Figure S3. Full-length agarose gel electrophoresis result of pET28a-TtAgo plasmid with different treatments, related to Figure 3C in the revised manuscript. Agarose gel was imaged with Gel Imager (MicroChemi, DNR) with 100 ms exposure time. Photoshop was used to add the label of molecular size markers and lane number.

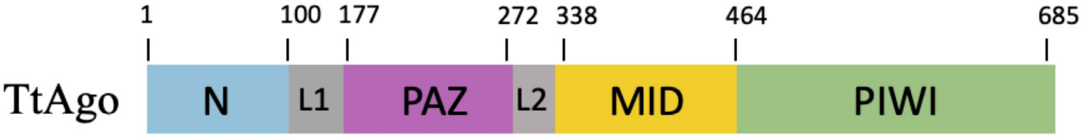

Figure S4. Linear representation of the 4 domains and 2 linkers of the TtAgo protein.

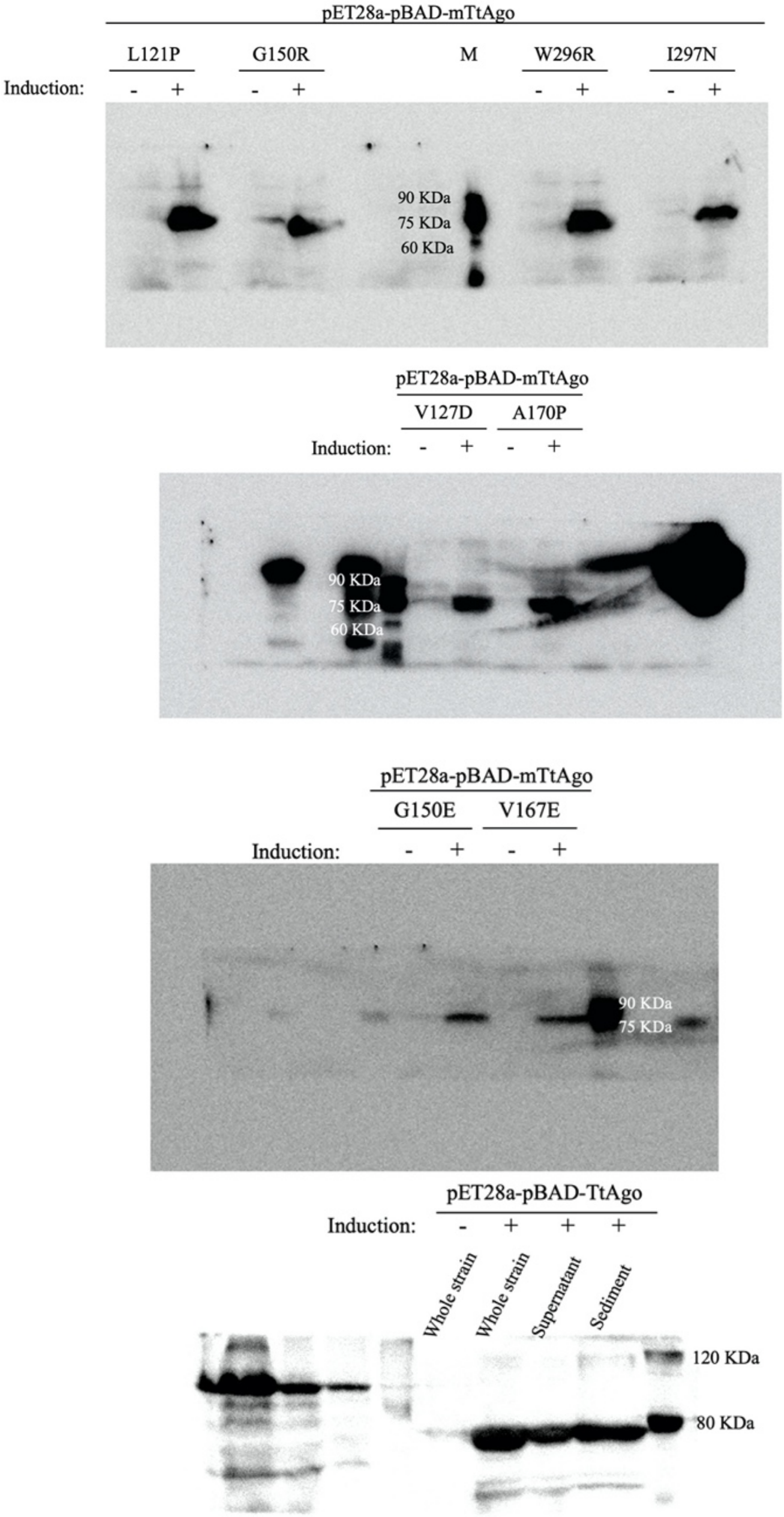

Figure S5. TtAgo and TtAgo mutant protein expression in BL21(DE3), related to Figure 9D in the revised manuscript. Induced (labeled as "+") and not induced (labeled as "-") cells from experiment shown on Fig. 9D were lysed and loaded on 10% SDS-PAGE gel followed by western blotting with anti-His antibody. The proteins were induced by arabinose of final concentration to 10 mM and with

a 6*His-tag on the N terminus. TtAgo wild type protein and TtAgo mutant protein were imaged with 1minute exposure time with ECL Western HRP substrate (Advansta) on a Fluorescent gel imaging system (MicroChemi, DNR) and the samples were derived from the same experiment and that blots were processed in parallel. PowerPoint was used to add the label of molecular size markers and samples.

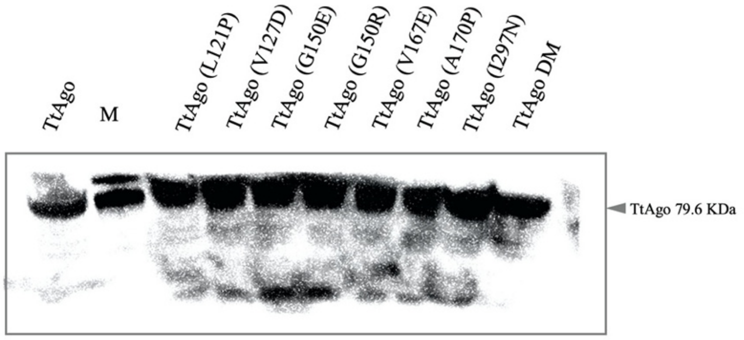

Figure S6. Full-length western blotting image of TtAgo and TtAgo mutant protein expression in DH5α, related to Figure 10B. Induced cells from experiment shown on Fig. 10B were lysed and loaded on 10% SDS-PAGE gel followed by western blotting with anti-His antibody. The proteins were induced by arabinose of final concentration to 10 mM and with a 6*His-tag on the N terminus. TtAgo wild type protein and TtAgo mutant protein were imaged with 15 seconds exposure time with ECL Western HRP substrate (Advansta) on a Fluorescent gel imaging system (MicroChemi, DNR) and the samples were derived from the same experiment and that blots were processed in parallel. PowerPoint was used to add the label of molecular size markers and samples.

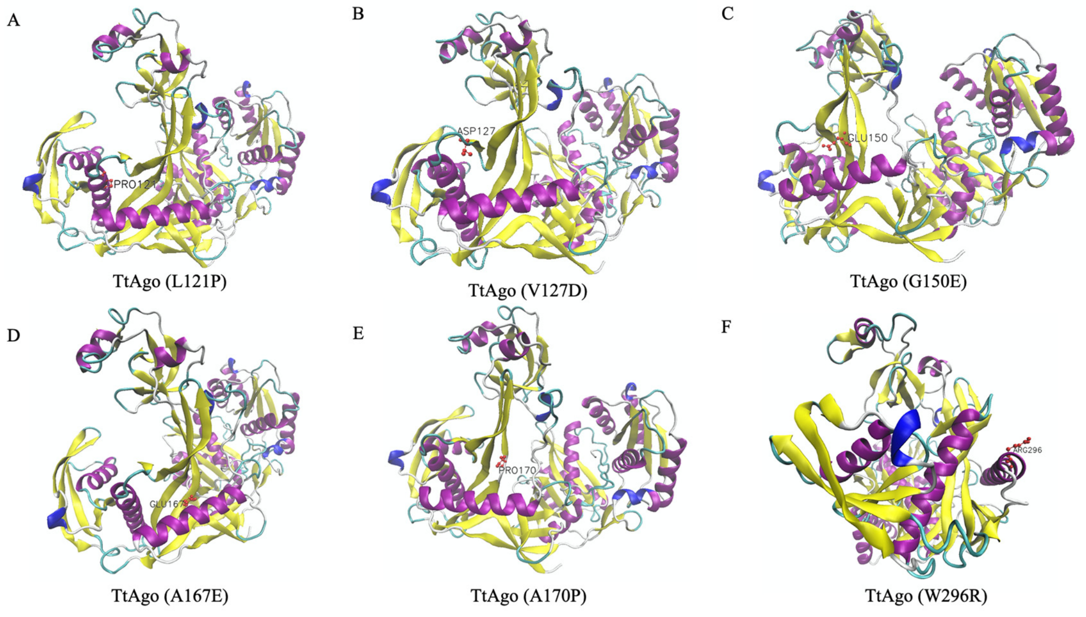

Figure S7. Protein structural simulation figures of the rest of the TtAgo mutants. (A) TtAgo (L121P); (B) TtAgo (V127D); (C) TtAgo (G150E); (D) TtAgo (A167E); (E) TtAgo (A170P); (F) TtAgo

(W296R). The figures were drawn in newcartoon method and colored by secondary structure, with alpha helix indicated in purple, beta-sheet in yellow and amino acids colored in CPK style.

Table S1. Primers used in this experiment

Primer name	Sequence (5'-3')
*Bgl*II-araC-R	ca*AGATCT*CCCCGAAAAGTGCC
*Xba*I-pBAD-R	tgc*TCTAGA*GAATTCCCAAAAAA
*Eco*RI-*Xba*I del-F	ccg*GAATTC*AATAATTTTGTTTAACTTTAAGAAG
*Nde*I-*Xba*I del-R	ggaattc*CATATG*GCTGCCGCGCGGCACCAG
*Sal*I-Tt-F	ggc*GTCGAC*GGACCCGGGAG
*Hin*dIII-*Xba*I-*Spe*I-Tt-R(del TAA)	ccc*AAGCTT*tgc*TCTAGA*ataagg*ACTAGT*cc GTGATGGTGATGGTGATGAA
*Spe*I-gsg link-fab1-F	cgg*ACTAGT*GGGTTCTGGCATGGGTTTCTTTCCGG TAAGC
*Xba*I-fab1-R	cgg*TCTAGA*TTATTTCAGTTCGAGTTCGTTC
FabIG93V-overlap-F	GTACACTCTATTGTTTTTGCACCTGGC
FabIG93V-overlap-R	GCCAGGTGCAAAAACAATAGAGTGTAC
*Nde*I-Tt-F	ggaattc*CATATG*AACCACCTTGGAAAAACGGAAG
*Hin*dIII-6*His-Tt-R	CGC*AAGCTT*CTAGTGATGGTGATGGTGATGAACG AAGAAGAGCTTTTCCC
*Hin*dIII-TAA-TtV685-R	cccAAGCTTttaAACGAAGAAGAGCTTTTCCC
*Spe*I-Tt-R	cgg*ACTAGT*CCGTGATGGTGATG
Tt-F *Sal*I	gc*GTCGAC*GGACCCGGGAGATC
D478A overlap-F	CCGTGGGCTTTGcCGCTGGCGGAA
D478A overlap-R	TTCCGCCAGCGgCAAAGCCCACGG
D546A overlap-F	CCTCCTTCGGGcCGGCCGTGTG
D546A overlap-R	CACACGGCCGgCCCGAAGGAGG
Tt-R *Hin*dIII	cgc*AAGCTT*CTAGTGATGGTGATGGTGATG
A53E-F	AGGTGGCCCGCCGGGAGGGGGGC
A53E-R	GCCCCCCTCCCGGCGGGCCACCT
R59H-F	TCACGGTGCACATGGGAGACGGC
R59H-R	GCCGTCTCCCATGTGCACCGTGA
L75F-F	CCCCTGAGGTCCTGGTCTTTGAGGGCACCT
L75F-R	AAAGACCAGGACCTCAGGGGGCGACCAGGA
P97S-F	GGGAGGAGGTCTCTGGACCCCAAG
P97S-R	CTTGGGGTCCAGAGACCTCCTCCC
A113V-F	TTTCGGCCTTGGTCCGAAGGCTCC
A113V-R	GGAGCCTTCGGACCAAGGCCGAAA
L121P-F	CAGGAGCGCCCCAGGCGCCTCGAGGGG
L121P-R	CCCCTCGAGGCGCCTGGGGCGCTCCTG
V127D-F	CCTCGAGGGGGACTGGGTGGAG
V127D-R	CTCCACCCAGTCCCCCTCGA
Tt-*Sal*I-R	CTCCCGGGTCC*GTCGAC*GCC
G144R-F	GGGCCCAGGTGGCGGGTGCTTG
G144R-R	CAAGCACCCGCCACCTGGGCCC
L148P-F	GGGCCCGGGTGGCGGGTGCCTGGGGGGGCG

L148P-R	GGCACCCGCCACCCGGGCCCCCGGGCGTGC
G150R-F	GCTTGGGAGGGCGGTCTTGGAC
G150R-R	GTCCAAGACCGCCCTCCCAAGC
G150E-F	GGGTGCTTGGGGAGGCGGTCTTGGA
G150E-R	TCCAAGACCGCCTCCCCAAGCACCC
A151E-F	CTTGGGGGGGTGGTCTTGGACCTT
A151E-R	AAGGTCCAAGACCACCCCCCCAAG
V167E-F	CCTCCTGGAGGAGGACCCCGCTTAC
V167E-R	GTAAGCGGGGTCCTCCTCCAGGAGGAA
A170P-F	TCCTCCTGGAGGTGGACCCCCCTTACCGGATC
A170P-R	GGGGGTCCACCTCCAGGAGGAACGCCCCCGAG
A178T-R	TCCGTCGACGCCGCTCTTCCCAAGGCAAGGAGA GGGTCAGGCTT
L189Q-F	AAGGCCACCCTCAGCCCAAAC
L189Q-R	GTTTGGGCTGAGGGTGGCCTT
I293F-F	GCGTCGACGGACCCGGGAGTTCGCCAGCTG
I293F-R	ACTCCCGGGTCCGTCGACGCCGCTCTTCCC
W296R-F	GGCGTCGACGGACCCGGGAGATCGCCAGCCGGA TCGGC
I297N-F	GGCGTCGACGGACCCGGGAGATCGCCAGCTGGA ACGGC
R427M-F	TCTCCTCATGGAAGGCCTTCCCA
R427M-R	TGGGAAGGCCTTCCATGAGGAGA
L430P-F	TCAGGGAAGGCCATCCCAGCCAAA
L430P-R	TTTGGCTGGGATGGCCTTCCCTGA
R486 overlap-F	GGAAGGGAGTCCTTTCACTTCGGG
R486overlap-R	CCCGAAGTGAAAGGACTCCCTTCC
R545 overlap-F	CCTCCTCCTTCACGACGGCCGT
R545 overlap-R	CCCGAAGTGAAAGGACTCCCTTCC
L556P-F	AGTTCGCCCCGGCCTTGGAGGCC
L556P-R	GGCCTCCAAGGCCGGGGCGAACT
R651W-F	TCGCCTTCCCCTGGCTTCCCGCT
R651W-R	AGCGGGAAGCCAGGGGAAGGCGA
A659S-F	CTTCACCTGTCCGACCGCCTGGT
A659S-R	ACCAGGCGGTCGGACAGGTGAAG

Table S2. Error-prone PCR reaction system

Regent	Volume
Template DNA	50 ng
10x Taq buffer	5 µl
100 mM dGTP	0.1 µl
100 mM dATP	0.5 µl
100 mM dTTP	0.5 µl
100 mM dCTP	0.5 µl
Primer-F	1 µl
Primer-R	1 µl
50 mM $MgSO_4$	0/1/2/3/4/5 µl
50 mM $MnCl_2$	0.5 µl
Taq enzyme	0.5 µl
ddH_2O	
∑	Up to 50 µl
